# Modifications in Prefrontal Cortex Oxygenation in Linear and Curvilinear Dual Task Walking: A Combined fNIRS and IMUs Study

**DOI:** 10.3390/s21186159

**Published:** 2021-09-14

**Authors:** Valeria Belluscio, Gabriele Casti, Marco Ferrari, Valentina Quaresima, Maria Sofia Sappia, Jörn M. Horschig, Giuseppe Vannozzi

**Affiliations:** 1 Department of Movement, Human and Health Sciences, Interuniversity Centre of Bioengineering of the Human Neuromusculoskeletal System, University of Rome “Foro Italico”, Piazza Lauro de Bosis 15, 00135 Roma, Italy; valeria.belluscio@gmail.com (V.B.); gabriele.casti@gmail.com (G.C.); 2 IRCCS Santa Lucia Foundation, Via Ardeatina 306, 00179 Roma, Italy; 3 Department of Life, Health and Environmental Sciences, University of L’Aquila, 67100 L’Aquila, Italy; marco.ferrari@univaq.it (M.F.); valentina.quaresima@univaq.it (V.Q.); 4 Artinis Medical Systems B.V., 6662 PW Elst, The Netherlands; sofia@artinis.com (M.S.S.); science@artinis.com (J.M.H.); 5 Donders Institute for Brain, Behaviour and Cognition, Radboud University Nijmegen, 6525 EN Nijmegen, The Netherlands

**Keywords:** biomechanics, cortical activity, functional near-infrared spectroscopy, locomotion, curved walking, gait performance, inertial sensors, acceleration, gait quality, wearable technology

## Abstract

Increased oxygenated hemoglobin concentration of the prefrontal cortex (PFC) has been observed during linear walking, particularly when there is a high attention demand on the task, like in dual-task (DT) paradigms. Despite the knowledge that cognitive and motor demands depend on the complexity of the motor task, most studies have only focused on usual walking, while little is known for more challenging tasks, such as curved paths. To explore the relationship between cortical activation and gait biomechanics, 20 healthy young adults were asked to perform linear and curvilinear walking trajectories in single-task and DT conditions. PFC activation was assessed using functional near-infrared spectroscopy, while gait quality with four inertial measurement units. The Figure-of-8-Walk-Test was adopted as the curvilinear trajectory, with the “Serial 7s” test as concurrent cognitive task. Results show that walking along curvilinear trajectories in DT led to increased PFC activation and decreased motor performance. Under DT walking, the neural correlates of executive function and gait control tend to be modified in response to the cognitive resources imposed by the motor task. Being more representative of real-life situations, this approach to curved walking has the potential to reveal crucial information and to improve people’ s balance, safety, and life’s quality.

## 1. Introduction

Functional near-infrared spectroscopy (fNIRS), a non-invasive vascular-based neuroimaging technique [1,2], has revolutionized the field of gait and posture [3,4]. This measurement technique aims to assess cerebral blood flow that should be kept at an adequate level to support brain activity and metabolic demand, and that is maintained through the processes of neurovascular coupling. When a specific brain region is activated, cerebral blood flow increases in a temporally and spatially coordinated manner tightly linked to changes in neural activity through a complex sequence of events involving neurons, arteries/arterioles, and signaling molecules. The fNIRS relies on this coupling to infer changes in neural activity that are mirrored by the changes in the blood oxygenation in the region of the activated cortical area. The fNIRS enables real-time detection of changes in oxyhemoglobin (ΔO_2_Hb) and deoxyhemoglobin (ΔHHb) within cortical areas, while having low susceptibility to motion artifacts. Compared to the traditional brain imaging technique (i.e., functional magnetic resonance imaging, fMRI), in which the evaluation of dynamic tasks is limited due to the fixed lying position, wearable fNIRS allows the assessment of cortical function and a better understanding of motor-cognitive interactions during real-life locomotion [1].

The ability to walk is critical for functional independence: an efficient motor control relies on neuronal networks enclosing cortical brain structures [1,3], and continuous adjustments of sensory and cognitive systems are necessary in order to perform safe locomotion, adaptable to individual abilities and environmental burdens [4]. The prefrontal cortex (PFC) is considered part of the executive locomotor pathway: PFC and related circuits are, in fact, known to be involved in the cognitive control of walking, running, and balance in healthy individuals [5,6,7], older adults [8], and people with neurological diseases [9,10]. The execution of a concurrent cognitive task while performing a motor task, the so-called dual-task (DT) paradigm, further increases the cognitive demand and potentially results in a decrease in one or both tasks’ performance compared to when tasks are performed separately, as in single-task (ST) [11,12,13,14].

Nonetheless, cognitive and motor demands depend on the complexity of the motor task [15]: compared to linear walking, which is considered an automatic task [16], curvilinear walking represents a challenge for the motor control system [17] and it is used to replicate more of the complexity of walking in daily life than straight-path walking [18]. The Figure-of-8 Walk test (F8WT) has been commonly adopted to measure walking abilities necessary for independence [18,19]: F8WT requires walking along both straight and curved sections and involves steering the body in clockwise and counterclockwise directions. Fine coordination of body segments’ movements and body reorientation toward the new travel direction are, therefore, necessary [17]. In addition, reduced dynamic balance and body symmetry, as well as greater demands on balance control have been observed during the F8WT compared with straight-path walking, particularly in the mediolateral direction [16,18]. The complexity of the F8WT has also been used to investigate the role of the PFC [15]. Lower-limb amputees and healthy controls performed the F8WT in three different conditions: a usual walking, carrying a load, and walking on uneven terrain, reporting higher cortical activation during the most difficult walking modality [15]. However, almost no information has been provided by the authors about people’s walking patterns, which can provide a description of individuals’ gait quality: what happens when a concurrent cognitive task is carried out during a challenging motor task such as the F8WT? Is there a decrement in terms of temporal parameters and dynamic balance indices in the walking performance?

In order to answer to these questions and further investigate the role of the PFC in the locomotor pathway, the aim of this study was to measure PFC activation while performing linear and curvilinear walking trajectories, in ST and DT conditions. A correlation analysis was also performed to better understand the relationship between cortical activity and gait quality parameters. The hypothesis was that curvilinear paths would have increased PFC oxygenation more than linear trajectories, accompanied by a decrease in the walking performance, and this increased activation would be more evident in the DT condition.

## 2. Materials and Methods

The study was approved by the Institutional Review Board of University of Rome “Foro Italico” (approval number: CAR 57/2020). All participants provided their written, informed consent for participating in the study.

### 2.1. Participants

Twenty healthy, young adults were enrolled in the study. Demographic and anthropometric participant’s characteristics are reported in Table 1. This sample size complied with the minimum number of participants recommended by a power analysis purposely performed for non-parametric comparisons (effect size d = 0.7, power 1-β = 0.82, α = 0.05) [20]. To be eligible, subjects were required to (1) have an age between 18 and 44 years, limits included; (2) not have suffered from an injury or anything that could influence gait, in the 3 months prior the testing session; (3) not have had any symptoms and/or not have been tested positive for SARS-CoV-2 infection in the last 3 months; and (4) not have any motor and/or visual deficits (that cannot be corrected with lenses) that might influence the motor performance.

### 2.2. Protocol

Each participant was asked to perform a total of six trials, three in the ST condition (only motor task) and three in the DT condition (motor task + cognitive task) at their preferred walking speed, while wearing comfortable shoes. Each trial lasted for 30 s. Two motor tasks were administered to participants: a linear walking, which consisted in walking back and forth along a 14-m flat path walkway, and a curvilinear walking, represented by the F8WT, which consisted in walking along a 8-shape figure marked on the floor with tape (circle diameter: 1.66 m), as previously proposed by [18], in both clockwise and counterclockwise directions. The “Serial 7s” cognitive task (S7) consisted in serially subtracting 7 from a three-digit given number (i.e., 170, 163, 158, 151…) [5]. Counting backward performance was assessed by rate (enumerated numbers during the 30 s) and accuracy (percentage of correct answers). For each counting trial, a different number was given to participants at the starting point. In the case of a wrong answer, the right number was provided to the participant and the error was marked for further considerations. No motor or cognitive task priority instructions (i.e., give equal priority to both tasks or sequential execution of tasks) were given.

Each condition started and ended with 20 s of quiet standing, with the instruction to refrain from talking and/or moving the head. After these initial 20 s, the instruction “start” was given and participants were asked to walk (or walk + count, based on the instructions) for 30 s, until the “stop” signal. The quiet standing of 20 s before each task was collected as the baseline reference for PFC ΔO_2_Hb and ΔHHb, as described by [21,22]. The order in which the six walking conditions were performed was randomized.

### 2.3. The fNIRS Data Acquisition and Processing

PFC ΔO_2_Hb and ΔHHb were measured with the Brite24 fNIRS system (Artinis Medical Systems B.V., Elst, the Netherlands). The system uses near-infrared light, transmitted at two wavelengths: 760 and 850 nm. Data were sampled with a frequency of 10 Hz and transmitted via Bluetooth to a laptop for further analyses. The probe consisted of 20 measurement points (source-detector distance about 3.5 cm) mounted on a soft neoprene head cap, which covers the frontal cortex based on the 10–20 EEG map [23]. Based on different absorption spectra, concentration changes of PFC O_2_Hb and HHb were calculated from the changes in detected light intensity using the modified Lambert–Beer law, assuming constant scattering [24]. Oxysoft software (version 3.2.70) was used to obtain the signal and calculate the differential pathlength factor according to the participants’ age. Pre-processing of the fNIRS signal was completed with custom-written code (MATLAB^®^ 2015b, The MathWorks, Inc., Natick, MA, USA). A lowpass filter of 0.1 Hz was used to reduce physiological noise, such as heart beat and drift of the signal. To remove motion artifacts, a wavelet filter was used [25,26] as well as a principal component analysis (PCA) filter with the recommended value of 80% of explained variance to eliminate motion artifacts [26]. Finally, the filtered optical density signals were transformed into concentration changes of O_2_Hb and HHb. After the pre-processing, HRF of each channel was averaged into two regions of interest, left and right PFC. The first 5 s of the task were eliminated to take into account the delay in the hemodynamic reaction to a stimulus, while the remaining 25 s were normalized by subtracting the median value of the pre-stimulus baseline from the signal in order to remove the intraindividual variance of the starting value. As also reported in previous studies, ΔO_2_Hb seems to be a more reliable parameter for measuring mobility-dependent changes in cortical oxygenation than ΔHHb [3,6,14]: Therefore, ΔO_2_Hb is here reported as primary outcome for PFC activation.

### 2.4. IMUs Data Acquisition and Processing

A set of four automatically synchronized wearable inertial sensors (Opal, APDM, Portland, OR, USA, 128 Hz) was used. Each unit embedded three-axial accelerometers and gyroscopes (±6 g with g = 9.81 m/s^2^, ±1500°/s of full-range scale, respectively) and provided the quantities with respect to a unit-embedded frame. IMUs were placed at the center of the sternum, at the pelvis (L4–L5 level), and at both shins’ level, slightly above lateral malleoli. To guarantee a repeatable reference system for the IMUs located on the upper body, each unit was aligned with the corresponding anatomical axes (antero-posterior: AP, medio-lateral: ML, and cranio-caudal: CC) following the procedure proposed by [27]. All data processing was performed using Matlab^®^. Only steady-state strides were analyzed, excluding the first and the last two strides for each walking trial. Temporal parameters and, more specifically, average stride duration (SD = time to complete the test/ total number of strides) and average stride frequency (SF = total number of strides/time to complete the test) were obtained through a peak detection algorithm applied to the ML angular velocity signals measured by the two IMUs on the shanks. Average walking speed (WS) was obtained using a stopwatch and considering a straight 10-m path (10 m/time to complete the test) and an 8-shape path (8-shape/time to complete the test). The following gait quality indices related to dynamic stability of gait were estimated from upper body IMUs:

- Normalized Root Mean Square (nRMS), a measure of acceleration dispersion and computed as follows:

$$\mathrm{RMS}=\sqrt{\frac{\sum_{n-1}^{N}a_{i}}{N}}$$

where a_i_ is the acceleration measured at pelvis and trunk level. To account for the influence of the walking speed on this parameter, RMS values were normalized according to [28].

- Attenuation Coefficients (AC) between pelvis (P) and sternum (S) for each acceleration component (j) [29], defined as:

$$\mathrm{ACPSj}=1-\frac{\mathrm{RMSj}\;S}{\mathrm{RMSj}\;P}$$

### 2.5. Statistical Analysis

Inferential statistical analysis was performed using the IBM SPSS Statistics software (v23, IBM Corp., Armonk, NY, USA; alpha level of significance = 0.05). The normal distribution of each parameter was verified using the Shapiro–Wilk test. Since the parameters were mostly not normally distributed, a Friedman Test was performed. For the post hoc analysis, the Wilcoxon Signed Rank test was used, and Holm–Bonferroni correction was adopted, to prevent type I error inflation due to multiple comparisons. Spearman correlation coefficient (Rho) was computed for assessing correlations between cortical and gait quality parameters.

## 3. Results

Temporal and gait quality parameters in F8WT in both clockwise and counterclockwise directions were averaged to improve readability since no statistically significant differences were found either in ST or in DT (*p* > 0.05). Therefore, comparisons were made among linear ST, linear DT, F8WT ST, and F8WT DT.

### 3.1. Rate and Accuracy of Counting Performance

**Figure 1:**
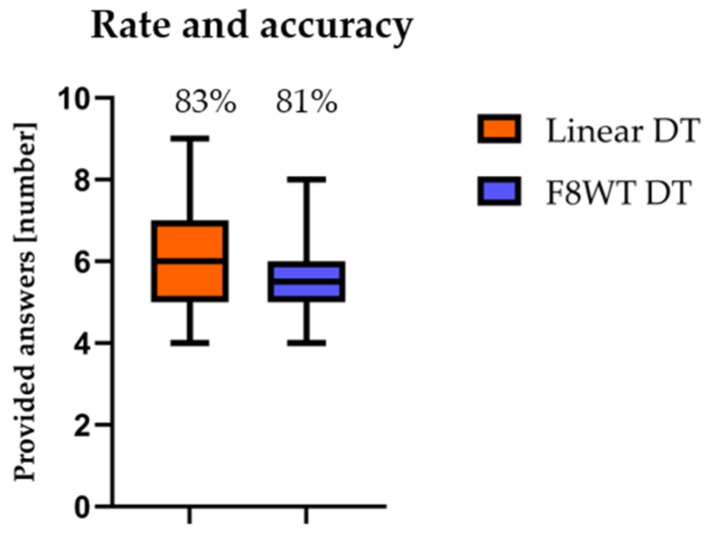
Whiskers’ plots: Enumerated numbers during the 30s of each task are reported; percentage at the top describes the accuracy of the given answers. DT = dual task; F8WT = Figure-of-8 Walk test.

Counting backward performance was assessed through rate (enumerated numbers during the 30 s) and accuracy (percentage of correct answers). Results are reported in Figure 1. No statistically significant differences were found during the S7 test (*p* > 0.05) between the different tasks either for rate (*p* = 0.291) or accuracy (*p* = 0.121).

### 3.2. The fNIRS Results

**Figure 2:**
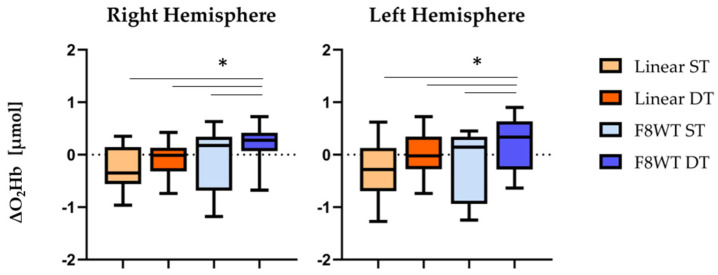
Median and interquartile ΔO_2_Hb values are reported. Horizontal lines and asterisks indicate significant statistical differences. ST = single task; DT = dual task; F8WT = Figure-of-8 Walk test * = *p* < 0.05.

Figure 2 reports ΔO_2_Hb values for right and left hemispheres. No statistically significant differences were observed when comparing the two hemispheres (*p* > 0.05). Interestingly, F8WT DT presented statistically significant differences both right and left hemispheres in the comparisons with the other three conditions: linear ST (*p* = 0.001 and Z = −3.659; *p* = 0.002 and Z = −3.173), linear DT (*p* = 0.001 and, Z = −3.248; *p* = 0.003 and Z = −2.917), and F8WT ST (*p* = 0.001 and Z = −3.211; *p* = 0.001 and Z = −3.654), respectively for right and left hemispheres.

### 3.3. IMUs’ Results

#### 3.3.1. Temporal Parameters

Table 2 reports the temporal parameters’ results. Statistically significant differences were found when considering the walking speed parameters in the comparisons between linear ST and F8WT ST (*p* = 0.003 and Z = −2.917) and linear ST and F8WT DT (*p* = 0.001 and Z = −3.211).

#### 3.3.2. Gait Quality indices

**Figure 3:**
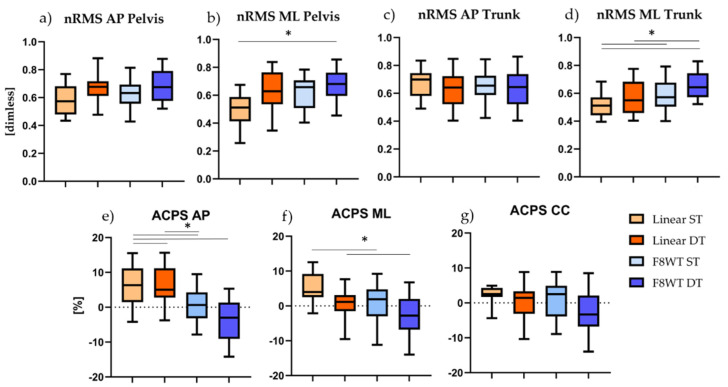
Median and interquartile values are displayed. Horizontal lines and asterisks indicate significant statistical differences. F8WT = Figure-of-8 Walk test; ST = single task; DT = dual task; nRMS = normalized root mean square; ACPS = attenuation coefficient pelvis-sternum; AP = antero-posterior; ML = medio-lateral; CC = cranio-caudal.; * *p* < 0.05.

Normalized Root Mean Square and Attenuation Coefficients results are shown in Figure 3. Statistically significant differences were found when considering the nRMS ML at pelvis level for the comparison between linear ST and F8WT DT (*p* = 0.007 and Z = −2.688); the nRMS trunk ML for the comparisons between linear ST and F8WT DT (*p* = 0.002 and Z = −3.173), linear DT and F8WT DT (*p* = 0.001 and z = −3.211), and F8WT ST and F8WT DT (*p* = 0.002 and Z = −3.061); the ACPS AP for the comparisons between linear ST and linear DT (*p* = 0.009 and Z = −2.614), linear ST and F8WT ST (*p* = 0.007 and Z = −2.688), linear ST and F8WT DT (*p* = 0.012 and Z = −2.502), and linear DT and F8WT ST (*p* = 0.004 and Z = −2. 563); the ACPS ML for the comparisons between linear ST and F8WT ST (*p* = 0.003 and Z = −2.917) and linear DT and F8WT ST (*p* = 0.010 and Z = −2.567).

### 3.4. Correlation Analysis Results

**Figure 4:**
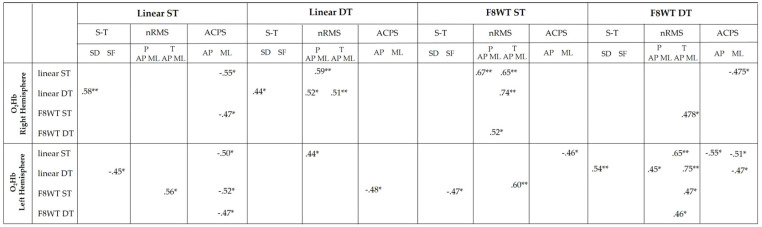
O_2_Hb = oxygenated hemoglobin; SF = stride frequency; SD = stride duration; F8WT = Figure-of-8 Walk test; ST = single task; DT = dual task; P = pelvis; T = trunk; S-T = spatio-temporals; nRMS = normalized root mean square; ACPS = attenuation coefficient pelvis-sternum; AP = antero-posterior; ML = medio-lateral; * = *p* < 0.05; ** = *p* < 0.01.

The results of the correlation analysis between PFC ΔO_2_Hb and gait quality parameters are reported in Figure 4.

## 4. Discussion

The aim of the study was to measure PFC activation while performing linear and curvilinear walking modalities, in ST and DT conditions. PFC cortical activation was assessed through an fNIRS device, while a wearable sensor-based protocol was adopted in order to obtain information about gait patterns. Results showed that (1) walking along curvilinear trajectories while performing a concurrent cognitive task led to increased PFC activation and decreased motor performance and (2) ΔO_2_Hb values in both right and left hemispheres were strongly correlated with several spatio-temporal and gait quality parameters in all performed conditions.

The cognitive task performance (Figure 1) was not affected by the type of walking trajectory: The number of subtractions completed was similar during linear and curvilinear walking, with an analogous number of mistakes. This result was also reported in a previous study [22], in which rate and accuracy of given answers in a S7 task were similar during walking and standing motor assessments. Conversely, our results contradict the findings in [30], in which young subjects were able to maintain appropriate attention to both calculation and stepping. However, the calculating/stepping DT used in the above-mentioned study was probably not sufficiently challenging, as opposed to the F8WT, where a cognitive demand is already embedded in the task. These results seem to suggest that healthy young adults may turn their attention to the execution of the calculation task at the expense of the motor task when this latter is particularly demanding.

According to our initial hypothesis, findings related to O_2_Hb suggest that there is an increased requirement for PFC activity during walking along a curvilinear trajectory, and that this demand further increases in presence of a concurrent cognitive task. Consistently with previous studies [13,14,22,31], a DT activity imposes a greater cognitive load: The increased O_2_Hb concentration in the F8WT during the serial subtraction seems to reflect the cognitive demands required by the DT itself. As observed in [22], O_2_Hb was gradually increased from normal walking, to walking while counting forward, to walking while serially subtracting 7. Additionally, [15] found an increase in the O_2_Hb while walking on uneven terrain compared to usual walking and walking while carrying a tray with two cups filled with water. Our results seem to confirm that the complexity of the motor task influences the PFC activity, and this is even more evident when young adults are also asked to perform a concurrent cognitive task.

For what concerns the motor assessment, results not only highlight the difficulty in performing a curvilinear task with respect to a linear task, but also that there is a further decrement in the gait performance in the DT condition (Figure 3d–g). Several studies have shown a strong relationship between poor executive functioning and slower gait speed, especially during dual tasks that involve a challenging locomotor component [21,23,32]. In our study, curved walking challenged motor control mechanisms more than straight walking [18]: Along curvilinear trajectories, in fact, healthy young adults reduced their walking speed (Table 2), confirming that human subjects adapt their velocity to the radius of the curvature that they are following, with the velocity that tends to decrease when the trajectory becomes more curved [18,33].

Concerning gait quality indices, the ML direction seems to be the most affected by the curvilinear trajectory: Higher nRMS values were found at both pelvis and sternum levels (Figure 3b–d, respectively) when performing the F8WT compared to the linear walking. The increased sternum accelerations during curved walking caused a significant decrease of the attenuation coefficient (AC) from the pelvis to the sternum in the AP and ML directions (Figure 3e–f). Increased body accelerations, reflected by higher nRMS and reduced ACPS, have been frequently linked to decreased gait stability [29,34,35,36]: As also previously observed, walking along a curvilinear trajectory generated medio-lateral torques necessary to counteract the centrifugal acceleration, provoking a shifting of the body center of mass toward the inner part of the curve [18,37]. The different motor strategies adopted by our participants during the two motor tasks highlighted that several adaptations are needed in order to steer a curved path in a safe way. The concurrent execution of a DT further decreased the gait performance: This phenomenon can be explained by the cognitive interference caused by competing demands for limited attentional resources [38]. The performance of a motor task, in fact, can be negatively influenced by a concurrent cognitive task: Brain resources need to be shared between motor and cognitive tasks, but since parallel processing is not possible, the two different tasks compete for the same processing resources, and increased demands by one task reduce the number of available resources for the other task [38,39]. Consistently with the findings in [40], healthy subjects seem to prioritize the cognitive task with a consequent detriment of the motor performance.

Furthermore, several correlations were identified between cortical activity and gait performance parameters in both the right and left hemispheres (Figure 4). In particular, ΔO_2_Hb was strongly correlated with temporal and dynamic balance-related parameters: Increased O_2_Hb was positively correlated with the stride duration and the nRMS values, while negatively correlated with the stride frequency and the ACPS values. One explanation is that, despite the higher PFC activation that especially occurs when the task becomes more challenging, as along the curved-trajectory and in DT conditions, there is an inefficiency in the use of cognitive resources that is reflected in the reduced motor performance [23]. Since turning-related neural systems may be more vulnerable to functional impairments associated with neurological diseases [41], further investigations should be performed in order to observe possibly different cortical activations due to the neurological impairment.

Some limitations should be acknowledged: This study did not involve a comprehensive cognitive battery that could have been useful to uncover further cognitive relationships with cortical activity. In addition, we did not perform a baseline condition consisting in only performing the cognitive task. However, this study provided evidence that under DT walking the neural correlates of executive function and gait control tend to be modified in response to the cognitive resources imposed by the motor task, supporting evidence of a connection between motor and cognitive function during walking in complex situations.

## 5. Conclusions

In conclusion, healthy young adults showed an increased PFC activation while performing curvilinear motor tasks. This activation was even higher if a concurrent cognitive task was executed. Since the cognitive task performance was not affected, we can speculate that participants turned their attention to the execution of the cognitive task at the expense of the motor task. This is reflected in a decrement of the motor performance, as highlighted by temporal and dynamic balance parameters. Being more representative of real-life situations, this approach to curved walking should be further investigated in populations with neurological deficits: Slight deviations from normal walking, which are not easily detectable with the traditional clinical scales or straight walking motor tasks, can be, in fact, detected with the F8WT, as previously reported in the literature and confirmed by our results. In addition, curvilinear walking could be adopted for the early-stage detection of a disease through the sensor-based identification of subtle signs or symptoms. Nonetheless, a more complex task is a challenge for the musculoskeletal system that has the potential to reveal crucial information and to improve people’s balance, safety, and quality of life.

## Figures and Tables

**Table 1:** Demographic and anthropometric participant’s characteristics. Mean and standard deviations (in brackets) values are reported. Gender and hand-dominant side are expressed in percentage.

Participant’s Characteristics	
Sex [female-male %]	50-50
Age [years]	28.4 (5.1)
Body Mass [kg]	73.5 (12.1)
Height [cm]	174.9 (9.1)
Physical Activity [days/week]	2.9 (2.4)
Hand Dominant Side [right %]	87

**Table 2:** Median and interquartile ranges of temporal parameters are reported. WS = walking speed; SF = stride frequency; SD = stride duration; F8WT = Figure-of-8 Walk test; ST = single task; DT = dual task; * = statistically significant difference between linear ST and F8WT DT; ^§^ = statistically significant difference between linear ST and F8WT ST.

	Linear ST	Linear DT	F8WT ST	F8WT DT
**WS** [m/s]	1.31 ± 0.02 *^§^	1.27 ± 0.31	1.19 ± 0.28 ^§^	1.17 ± 0.08 *
**SF** [stride/s]	0.73 ± 0.09	0.71 ± 0.09	0.70 ± 0.13	0.70 ± 0.08
**SD** [s]	1.32 ± 0.15	1.31 ± 0.11	1.31 ± 0.11	1.36 ± 0.14

## Data Availability

The data presented in this study could be made available upon request to the corresponding author.

## References

[1] Menant J.C., Maidan I., Alcock L., Al-Yahya E., Cerasa A., Clark D.J., de Bruin E.D., Fraser S., Gramigna V., Hamacher D. (2020). A consensus guide to using functional near-infrared spectroscopy in posture and gait research. Gait Posture.

[2] Quaresima V., Ferrari M. (2019). A Mini-Review on Functional Near-Infrared Spectroscopy (fNIRS): Where Do We Stand, and Where Should We Go?. Photonics.

[3] Herold F., Wiegel P., Scholkmann F., Thiers A., Hamacher D., Schega L. (2017). Functional near-infrared spectroscopy in movement science: A systematic review on cortical activity in postural and walking tasks. Neurophotonics.

[4] Al-Yahya E., Dawes H., Smith L., Dennis A., Howells K., Cockburn J. (2011). Cognitive motor interference while walking: A systematic review and meta-analysis. Neurosci. Biobehav. Rev..

[5] Miyai I., Tanabe H.C., Sase I., Eda H., Oda I., Konishi I., Tsunazawa Y., Suzuki T., Yanagida T., Kubota K. (2001). Cortical mapping of gait in humans: A near-infrared spectroscopic topography study. Neuroimage.

[6] Suzuki M., Miyai I., Ono T., Oda I., Konishi I., Kochiyama T., Kubota K. (2004). Prefrontal and premotor cortices are involved in adapting walking and running speed on the treadmill: An optical imaging study. Neuroimage.

[7] Udina C., Avtzi S., Durduran T., Holtzer R., Rosso A.L., Castellano-Tejedor C., Perez L.-M., Soto-Bagaria L., Inzitari M. (2020). Functional Near-Infrared Spectroscopy to Study Cerebral Hemodynamics in Older Adults During Cognitive and Motor Tasks: A Review. Front. Aging Neurosci..

[8] Lim S.B., Louie D.R., Peters S., Liu-Ambrose T., Boyd L.A., Eng J.J. (2021). Brain activity during real-time walking and with walking interventions after stroke: A systematic review. J. Neuroeng. Rehabil..

[9] Kahya M., Moon S., Ranchet M., Vukas R.R., Lyons K.E., Pahwa R., Akinwuntan A., Devos H. (2019). Brain activity during dual task gait and balance in aging and age-related neurodegenerative conditions: A systematic review. Exp. Gerontol..

[10] Pelicioni P.H.S., Tijsma M., Lord S.R., Menant J. (2019). Prefrontal cortical activation measured by fNIRS during walking: Effects of age, disease and secondary task. PeerJ.

[11] Yogev-Seligmann G., Hausdorff J.M., Giladi N. (2008). The role of executive function and attention in gait. Mov. Disord..

[12] Lin M.I.B., Lin K.H. (2016). Walking while performing working memory tasks changes the prefrontal cortex hemodynamic activations and gait kinematics. Front. Behav. Neurosci..

[13] Holtzer R., Mahoney J.R., Izzetoglu K., Onaral B., Verghese J. (2011). fNIRS Study of Walking and Walking while Talking in Young and Old Individuals. J. Gerontol. Ser. A Boil. Sci. Med. Sci..

[14] Schack J., Pripp A.H., Mirtaheri P., Steen H., Güler E., Gjøvaag T. (2020). Increased prefrontal cortical activation during challenging walking conditions in persons with lower limb amputation–an fNIRS observational study. Physiother. Theory Pract..

[15] Courtine G., Schieppati M. (2004). Tuning of a Basic Coordination Pattern Constructs Straight-Ahead and Curved Walking in Humans. J. Neurophysiol..

[16] Godi M., Giardini M., Schieppati M. (2019). Walking along curved trajectories. Changes with age and Parkinson’s disease. Hints to rehabilitation. Front. Neurol..

[17] Belluscio V., Bergamini E., Tramontano M., Formisano R., Buzzi M.G., Vannozzi G. (2020). Does Curved Walking Sharpen the Assessment of Gait Disorders? An Instrumented Approach Based on Wearable Inertial Sensors. Sensors.

[18] Hess R.J., Brach J.S., Piva S.R., VanSwearingen J.M. (2010). Walking Skill Can Be Assessed in Older Adults: Validity of the Figure-of-8 Walk Test. Phys. Ther..

[19] Cohen J. (1992). Statistical power analysis. Curr. Dir. Psychol. Sci..

[20] Al-Yahya E., Johansen-Berg H., Kischka U., Zarei M., Cockburn J., Dawes H. (2016). Prefrontal cortex activation while walking under dual-task conditions in stroke: A multimodal imaging study. Neurorehabil. Neural Repair.

[21] Maidan I., Nieuwhof F., Bernad-Elazari H., Reelick M.F., Bloem B.R., Giladi N., Deutsch J.E., Hausdorff J.M., Claassen J.A.H., Mirelman A. (2016). The role of the frontal lobe in complex walking among patients with Parkinson’s disease and healthy older adults: An fNIRS study. Neurorehabil. Neural Repair.

[22] Mirelman A., Maidan I., Bernad-Elazari H., Nieuwhof F., Reelick M., Giladi N., Hausdorff J.M. (2014). Increased frontal brain activation during walking while dual tasking: An fNIRS study in healthy young adults. J. Neuroeng. Rehabil..

[23] Mirelman A., Maidan I., Bernad-Elazari H., Shustack S., Giladi N., Hausdorff J.M. (2017). Effects of aging on prefrontal brain activation during challenging walking conditions. Brain Cogn..

[24] Yücel M.A., Lühmann A.v., Scholkmann F., Gervain J., Dan I., Ayaz H., Boas D., Cooper R.J., Culver J., Elwell C.E. (2021). Best practices for fNIRS publications. Neurophotonics.

[25] Cooper R.J., Selb J., Gagnon L., Phillip D., Schytz H.W., Iversen H.K., Ashina M., Boas D.A. (2012). A systematic comparison of motion artifact correction techniques for functional near-infrared spectroscopy. Front. Neurosci..

[26] Brigadoi S., Ceccherini L., Cutini S., Scarpa F., Scatturin P., Selb J., Gagnon L., Boas D.A., Cooper R.J. (2014). Motion artifacts in functional near-infrared spectroscopy: A comparison of motion correction techniques applied to real cognitive data. Neuroimage.

[27] Bergamini E., Ligorio G., Summa A., Vannozzi G., Cappozzo A., Sabatini A.M. (2014). Estimating Orientation Using Magnetic and Inertial Sensors and Different Sensor Fusion Approaches: Accuracy Assessment in Manual and Locomotion Tasks. Sensors.

[28] Iosa M., Bini F., Marinozzi F., Fusco A., Morone G., Koch G., Cinnera A.M., Bonnì S., Paolucci S. (2016). Stability and Harmony of Gait in Patients with Subacute Stroke. J. Med. Biol. Eng..

[29] Mazzà C., Iosa M., Pecoraro F., Cappozzo A. (2008). Control of the upper body accelerations in young and elderly women during level walking. J. Neuroeng. Rehabil..

[30] Ohsugi H., Ohgi S., Shigemori K., Schneider E.B. (2013). Differences in dual-task performance and prefrontal cortex activation between younger and older adults. BMC Neurosci..

[31] Doi T., Makizako H., Shimada H., Park H., Tsutsumimoto K., Uemura K., Suzuki T. (2013). Brain activation during dual-task walking and executive function among older adults with mild cognitive impairment: A fNIRS study. Aging Clin. Exp. Res..

[32] Salzman T., Vallejo D.T., Polskaia N., Michaud L., St-Amant G., Lajoie Y., Fraser S. (2021). Hemodynamic and behavioral changes in older adults during cognitively demanding dual tasks. Brain Behav..

[33] Vieilledent S., Kerlirzin Y., Dalbera S., Berthoz A. (2001). Relationship between velocity and curvature of a human locomotor trajectory. Neurosci. Lett..

[34] Belluscio V., Bergamini E., Tramontano M., Bustos A.O., Allevi G., Formisano R., Vannozzi G., Buzzi M.G. (2019). Gait Quality Assessment in Survivors from Severe Traumatic Brain Injury: An Instrumented Approach Based on Inertial Sensors. Sensors.

[35] Bergamini E., Iosa M., Belluscio V., Morone G., Tramontano M., Vannozzi G. (2017). Multi-sensor assessment of dynamic balance during gait in patients with subacute stroke. J. Biomech..

[36] Kavanagh J.J., Menz H. (2008). Accelerometry: A technique for quantifying movement patterns during walking. Gait Posture.

[37] Turcato A.M., Godi M., Giordano A., Schieppati M., Nardone A. (2015). The generation of centripetal force when walking in a circle: Insight from the distribution of ground reaction forces recorded by plantar insoles. J. Neuroeng. Rehabil..

[38] Navon D., Miller J. (2002). Queuing or Sharing? A Critical Evaluation of the Single-Bottleneck Notion. Cogn. Psychol..

[39] Kahneman D. (1975). Attention and Effort.

[40] Mori T., Takeuchi N., Izumi S.-I. (2018). Prefrontal cortex activation during a dual task in patients with stroke. Gait Posture.

[41] Crenna P., Carpinella I., Rabuffetti M., Calabrese E., Mazzoleni P., Nemni R., Ferrarin M. (2007). The association between impaired turning and normal straight walking in Parkinson’s disease. Gait Posture.

